# DELAY on first antenatal care visit and its associated factors among pregnant women in public health facilities of Debre Markos town, North West Ethiopia

**DOI:** 10.1186/s12884-018-1748-7

**Published:** 2018-05-16

**Authors:** Atsede Alle Ewunetie, Alemtsehay Mekonnen Munea, Belsity Temesgen Meselu, Muluye Molla Simeneh, Bekele Tesfaye Meteku

**Affiliations:** 1 Department of Public Health, College of Health Sciences, Debre Markos University, Debre Markos, Ethiopia; 2 Department of Public Health, College of Medicine and Health Science, Bahir Dar University, Bahir Dar, Ethiopia; 3 Department of Midwifery College of Health Sciences, Debremarkos University, Debre Markos, Ethiopia; 4 Department of Nursing, College of Health Sciences, Debre Markos University, Debre Markos, Ethiopia

**Keywords:** Delay on timely initiation of antenatalcare, Unintended, Pregnancy

## Abstract

**Background:**

Delay on timely initiation of antenatal care has a great impact on adverse pregnancy out comes. However, evidences in Ethiopia revealed that majority of pregnant mothers did not start their first visit as recommrnded by WHO. The aim of this study was to assess delay and associated factors of first antenatal care visit among pregnant mothers at public health facilities of Debremarkos town, North West Ethiopia.

**Methods:**

An institutional based crosss-sectional study was conducted from February to March, 2014 in public health facilities of Debremarkos town North west Ethiopia. A total of 320 pregnant mothers who were sure of their last menstrual periods were interviewed with a structured questionnaire. Data entry was done using Epi data 3.1 and analysis was done using SPSS version 20. Descriptive statistics, binary and multivariable logistic regression analyses were employed to identify the magnitude and factors associated with delay on timely initiation of the first antenatal care visit.

**Results:**

The proportion of respondents who made their first antenatal care visit after 16 weeks of gestation was found to be 33.4%. Mothers residing in rural settings (AOR = 2.8 [95% CI:1.54–5.44]), not attained formal education(AOR = 2.2 [95% CI:1.10–4.68]),with unintended pregnancy (AOR = 3.6 [95% CI:2.00–6.80]) and who perceived that the right initiation time of the first antenatal care visit is beyond 16 weeks of gestation (AOR = 3.9 [95% CI:1.61–9.76]) were more likely delayed on their first antenatal care visit .

**Conclusion:**

Residence**,** educational status, intention of pregnancy and perception on the right time of first antenatal care visit initiation were found to be predictors of delay on timely initiatin of first antenatal care visit. Therefore, the Zonal health department should strengthen awareness creation about timely initiation of first antenatal care visit and family planning to prevent unintended pregnancy in the community especially in the rural settings.

## Background

Many health problems of pregnant women are preventable, detectable, and treatable if they attained antenatal care (ANC) adequately [1]. The traditional approach of the ANC is a high risk approach which intended to classify pregnant women at low risk or high risk based on predetermined criteria and involved many ANC visits. This approach was hard to implement effectively since many women had at least one risk factor, and not all developed complications; at the same time, some low risk women did develop complications. It is a burden on the healthcare system. As a result, many developing countries, including Ethiopia, are adopting focused antenatal care (FANC) approach. The newly proposed, FANC recommends four ANC visits for most pregnant women. Ideally, the first visit is in the first trimester but not after 16 weeks of gestation [2, 3].

The recommendation sets out from the fact that first trimester pregnancy stage is the fastest developmental period of the fetus, in which all organs become well developed and needs special attention [4, 5]. However, too many women make their first antenatal visit with the pregnancy already compromised or at risk from smoking, inappropriate nutrition, ingestion of a variety of drugs, including pharmaceutical preparations, genitourinary tract infections, anemia and poor dental health [5].

Women present for antenatal care early in their pregnancy period allow enough time for essential and feasible interventions, prevention of complications and early identification of underlying conditions [6]. It also used to prevent, diagnose and treat sexually transmitted infections and work on the elimination of new Human Immune Deficiency Virus infections among new borne through providing integrated quality prevention of mother to child transmission [7–9].

Early attendance of ANC provides a better hemoglobin concentration through nutritional advice, prevention and early treatment of malaria and timely iron foliate supplementation [10, 11]. Beside this, it increases the opportunity of pregnant mothers to have more prenatal care visits, sufficient tests and advice during pregnancy and a skilled birth attendant [12, 13].

Mothers who start ANC after 22 weeks gestation, missed over four routine antenatal visits, who did not seek care or who concealed their pregnancy were manifest 17% of maternal deaths [14]. Suboptimal antenatal care was also found to be the major contributory factor for still birth in India [15]. Mothers who sought antenatal care before the end of the third month had infants who weighed heavier compared to the infants of mothers who sought care later [11, 16, 17].

Even though there is improvement on antenatal care coverage and the World Health Organization recommendation is initiating ANC visit in the first trimester, the time of initiation of first ANC visit is varied throughout the world. In the Ethiopian context, it is recommended that the first ANC visit should be ideally taken place before 16 weeks of pregnancy (2). In order to improve maternal health care service utilization; all governmental health institutions of Ethiopia are providing focused antenatal care service for all pregnant mothers who come to the health institutions free of charge. Early initiation of antenatal care is promoted by health extension workers and health professionals in both urban and rural Keble’s. Beside women developmental armies who are delegated in the community have their own role in community mobilization regarding to antenatal care service utilization.

According to the Ethiopia demographic, health survey, over 34% of pregnant women were attending antenatal care at least once. But, only 19% had four or more antenatal care visits during their entire pregnancy, and 11% of women made their first antenatal care visit before the fourth month of pregnancy nationwide [3]. In order to improve maternal and child health, identifying timing of first antenatal care visit among pregnant mothers and factors that affect initiation of first ANC visit is paramount. This study is designed to assess delay on first antenatal care visit among pregnant mothers and factors that affect initiation of first ANC.

## Methods

### Study design and data sources

An institutional based cross sectional study design was employed from February 1st through March 30, 2014 in public health facilities of Debremarkos town. Debremarkos town is the capital of East Gojjam Zone, which is located 300 km North West of Addis Ababa and 265 km away from regional capital Bahir Dar.

The study populations were pregnant women who visited public health facilities of Debremarkos town for antenatal care and randomly selected during the study period. Those pregnant mothers who were seriously ill or not aware their menstrual period were excluded from the study. The sample size was determined using single population proportion formula with the following assumptions: the proportion of delayed ANC is 74% from previous study conducted in Debre Birhan (18), maximum acceptable marginal error of 5%, an alpha level of 0.05 and a none response rate 10% were used to obtain a sample size of 326.

All public health facilities (one referral Hospital and three health centers) in Debremarkos town were included in the study. The total sample size was proportionally allocated to health facilities based on average monthly flow of pregnant mothers for ANC in each heath facility. The study subjects were recruited by using systematic random sampling technique after identification of the first study subject by simple random sampling method. Multiple enumerations due to referral were avoided using filtering questions.

The outcome variable of the study was delay on initiation of first ANC visit and the explanatory variables included Socio-demographic factors (age, religion, ethnicity, marital status, educational status, average monthly income**),** Obstetric factors (parity, History of obstetric complication, history of Previous ANC visit and intention of pregnancy),Enabling factors (accessibility of information about ANC and interaction with healthprofessional**)** and Reinforcing factors (perception of mothers on the advantage of the ANC, timing of ANC visit and frequency of the ANC; reasons that initiate mothers to start ANC after 16 weeks, a decision made to seek ANC, intention of partner to watrds ANC service utilization).

Delay on initiation of first antenatal care visit is initiation of first ANC visit in public health facilities which have skilled health personnel after 16 weeks of gestation.

Data were collected using structured interviewer administered questionnaire adopted from literatures and contextualized to the local situations and study objectives. The data collection tool was translated into the local language (Amharic) and pretested on 5% of the actual sample size out of the study area. Four diploma nurses and one public health professional were involved as data collector and supervisor respectively after taking 1 day training.

### Ethical considerations

The study was conducted after obtaining ethical clearance from Bahir Dar University, College of medicine and Health sciences, Research Ethics Committee and letter of support from Amhara regional health bureau ethical review committee. Formal Permission paper was given to woreda health office, Debremarkos Referal Hosipital and responsible persons in each health institution accordingly. Verbal informed consent also obtained from the study participants after explaining the purpose of study. Participants were informed on their full right to skip any question or terminate their participation at any stage. Participants were also assured that there will be no harm or benefit of being participating in this study. All the information from the respondents was kept confidential.

### Data processing and analysis

The data were cleaned, coded and entered in Lauritsen JM, Bruus M, Myatt M. Epi Data.A comprehensive tool for validated entry and documentation of data. 2003; 3 and transferred to IBM SPSS Statistics for Windows, Version 20.0. Armonk, NY: IBM Corp for analysis. Both descriptive and inferential statistics were used to summarize the data. Those variables found to be statistically significant (*p* < 0.2) in the binary logistic regression analyses were entered into the multivariable logistic regression model and statistical significance was considered at *p* < 0.05.The strength of association was assessed by odds ratio (OR) with 95% confidence interval.

## Results

The response rate was 98.1%.The mean (±SD)age of respondents was 26.3(± 5.1).Among all respondents, 311 (97.2%) were Amhara in ethnic group and majority 299(93.4%) of mothers were orthodox Christian followers (Table 1).

**Table 1:** Socio demographic characterstics of pregnant mothers who were attending ANC in the public health facilities of Debremarkos town, Feburary-March 2014(*n* = 320)

Varaible		Frequency	Percent
Residence	Rural	69	21.6
	Urban	251	78.4
Age	15–19	16	5.0
	20–24	104	32.5
	25–29	121	37.8
	30–34	55	17.2
	≥35	24	7.5
Ethinicity	Amhara	311	97.2
	Others	9	2.8
Relegion	Orthodox chrstian	299	93.4
	Muslim /Protestant	21	6.6
Marital status	Never married	14	4.4
	Married	293	91.6
	Divorced/widowed	13	4
Educational status	Have no formal education	98	30.6
	Primary education	51	15.9
	Secondary education	87	27.2
	Tertiary education	84	26.3
Occupation	Government employee	81	25.3
	House wife	49	15.3
	Merchant	118	36.9
	Farmer	42	13.1
	Daily labourer	17	5.3
	Students / depende on family	13	4.1
Monthly income	< 1000 ETB(<Q1)	124	38.8
	1000–2987.50ETB	116	36.2
	> 2987.50ETB(>Q3)	80	25.0

Others = Oromo/Tigrie/Agew

**Fig. 1:**
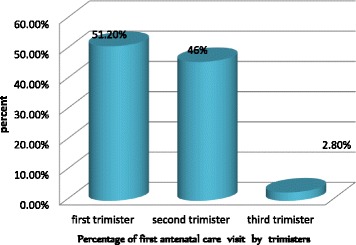
Distribution of first ANC visits among pregnant mothers in public health facilities in Debremarkos in each trimester between February 2014 and March 2014

Of all respondents, 107 (33.4%) made their first ANC visit after 16 weeks of gestation. The mean time was 14.5 (± 6.5) weeks with the range of 4 to 36 weeks. The median and the pick time was 12 weeks of gestation. Above half (51.2%) of mothers were start their first ANC visit in the first trimester (Fig. 1) .

Among the total respondents,174 (54.4%) were multiparous. Of these, 126 (74.2%) reported that they had had experience of ANC for the preceding pregnancy. For those who had had ANC visit on preceding pregnancy, 43 (34%) visited the ANC clinic for the first time after 16 weeks of gestation in the current pregnancy. From 68 (21.2%) of mothers who had unintended pregnancy, 25 (36.8%) indicated that they had interest in interrupting their pregnancy (Table 2).

**Table 2:** Obstetric history of pregnant mothers, who were attending ANC in the public health Facilities of Debre Markos town, Feburary – March, 2014 (*n* = 320)

Variable		Frequency	Percent
Gravidity (*n* = 320)	Primigravida	146	45.6
	Multi gravida	174	54.4
Parity (n = 320)	No parity	158	49.4
	≥1 parity	162	50.6
Children born alive (*n* = 174)	0	17	9.8
	1–3	135	77.6
	≥4	22	12.6
Death of children (*n* = 157)	Yes	30	19.1
	No	127	80.9
spontanous abortion (n = 174)	Yes	35	20.1
	No	139	79.9
stillbirth (*n* = 174)	Yes	18	10.3
	No	156	89.7
History of ANC (n = 174)	Yes	126	72.4
	No	48	27.6
ANC initiation time onPrevious pregnancy (*n* = 126)	≤16 Weeks	115	91.3
	> 16 Weeks	11	8.7
Type of pregnancy N = 320	Planned	252	78.8
	Unplanned	68	21.2
Intended to intrupt unplannedPregnancy *N* = 68	Yes	25	36.8
	No	43	63.2

The available health facilities were health centers for 220 (68.8%) of respondents, Hospital for 60(10.8%) of respondents and health posts for 40(12.4%) of respondents. Of all respondents, 182 (56.9%) were advised for ANC on current pregnancy. Of these, only 38 (20.9%) had information on the time of first ANC visit. From those who had information on initiation time of ANC, 2 (5.3%) were informed to start their first visit after 16 weeks of gestation (Table 3).

**Table 3:** Availablity and accessability of health information among mothers who were attending ANC in the public health facilities of Debremarkos town, Feburary-March 2014 (*n* = 320)

Variable		Frequency	Percent
Health education about ANCon previous pregnancy(n = 126)	Yes	85	67.5
	No	41	32.5
Advised for ANC (*n* = 320)	Yes	182	56.9
	No	138	43.1
By whom you were advised (*n* = 182)	Husbands	105	57.7
	HealtrhextentionWorkers	28	15.4
	Relatives	20	11
	Friends/nigbours	29	15.9
Advised on initiation time (*n* = 128)	Yes	38	20.9
	No	144	79.1
Advised time(*n* = 38)	≤16 weeks	36	94.7
	> 16 weeks	2	5.3

**Fig. 2:**
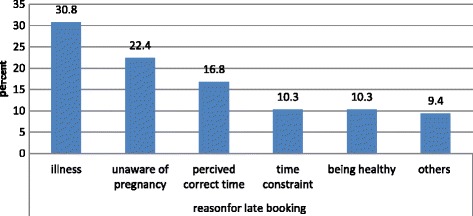
Reasons for delay on first ANC visit among pregnant mothers who attended ANC in the public health facilities of Debre markos town between February 2014 and March 2014

A majority, 27 (85.3%) of respondents perceived that the ANC is important for both maternal and child health. Among delayed respondents 18 (16.8%) were initiated ANC by considering the time of their visit at the right time (Fig. 2).

Among respondents 264 (82.5%) were decided to seek ANC service jointly with their partners.From all, 287(89.4%) partners supported the need of ANC for every pregnant mothers.

The bivariate analysis showed that pregnant mothers who were residing in rural areas[Crude Odds Ratio (COR) = 3.4, 95% Confidence Interval (CI): 2.01–6.07], not attained formal education[COR = 4.5,95% CI: 2.33–8.67], farmers [COR = 7.8, 95% CI: 3.37–18.05], having less than 1000 ETB average monthly income[COR = 2.7, 95% CI: 1.45–5.37],experience one or more births [COR = 1.9, 95% CI: 1.22–3.15], not planned their pregnancy [COR = 3.9, 95% CI: 2.25–6.89], having a history of stillbirth [COR = 2.6, 95% CI: 1.01–6.90] and those who perceived that the right time of first ANCvisit is after 16 weeks of gestation [COR = 3.8, 95% CI: 1.69–9.76] were more likely delayed on initiation of the first ANC visit.After adjusting for the potential cofounders; multivariable logistic regressionanalysis indicated that residence, educational status, intention of pregnancy and perception on the right time of first ANC visit were significantly associated with delayed first ANC visit.

Mothers who were residing in rural areas, not attainding formal education, having unplanned pregnancy and perciving the right time of ANC initiation as greater than 16 weeks of gestation were more likely delayed on first ANC visit (Adjusted Odds Ratio (AOR) =2.8 [95% CI:1.54–5.44]), (AOR = 2.2 [95%CI:1.10–4.68]),(AOR = 3.6 [95% CI: 2.00–6.80]) and (AOR = 3.9[95%CI:1.61–9.76]) respectivelly (Table 4).

**Table 4:** Factors associated with delay on first ANCvisit among pregnant mothers who were attending ANC in public health institutions of Debremarkos town,2014(n = 320)

Variable		Delayed		COR(CI)	AOR(C I)	*P*-Value
		Yes (%)	No (%)			
Residence						
	Rular	39(12.2)	30(9.4)	3.4(2.01–6.07)	2.8(1.54–5.44)^a^	0.001
	Urban	68(21.2)	183(57.2)	1.0	1.0	
Educational status						
	Had no formaleducation	54(16.9)	44(13.8)	4.5(2.33–8.67)	2.2 (1.10–4.68)^a^	0.027
	Primary education	16(5)	35(10.9)	1.6(0.76–3.68)	1.4(0.65–3.42)	
	Secondary education	19(5.9)	68(21.3)	1.0(0.49–2.12)	0.8(0.40–1.87)	
	Tertiary education	18(5.6)	66(20.6)	1.0	1.0	
Occupation						
	Government employee	18(5.6)	63(19.7)	1.0	1.0	
	Merchant	12(3.8)	37(11.6)	1.1(0.49–2.61)	0.9(0.30–2.98)	
	House wife	34(10.6)	84(26.3)	1.4(0.73–2.73)	1.0(0.37–2.94)	
	Farmer	29(9.1)	13(4.1)	7.8(3.37–18.05)	2.2(0.52–9.51)	
	Daily labourer	9(2.8)	8(2.5)	3.9(1.32–11.67)	1.5(0.33–7.21)	
	Student / dependonfamily	5(1.6)	8 (2.5)	2.1(0.63–7.51)	1.1(0.22–5.69)	
Monthly Income						
	< 1000 ETB	51(15.9)	73(22.8)	2.7(1.45–5.37)	1.5(0.66–3.59)	
	1000–2987.50 ETB	40(12.5)	76(23.8)	2.1(1.07–4.10)	1.4(0.66–3.30)	
	> 2987.50 ETB	16(5)	64(20)	1.0	1.0	
Parity						
	No parity	41(12.8))	117(36.6)	1.0	1.0	
	≥1 parity	66(20.6)	96(30)	1.9(1.22–3.15)	1.4(0.86–2.56)	
Types of pregnancy						
	Planned	67(20.9)	185(57.8)	1.0	1.0	
	Unplanned	40(12.5)	28(8.8)	3.9(2.25–6.89)	3.6(2.00–6.80)^a^	0.000
Histrory of still birth						
	No still birth	97(30.3)	205(64.1)	1.0	1.0	
	≥ 1 still birth	10 (3.1)	8(2.5)	2.6(1.01–6.90)	1.6(0.54–5.10)	
Perceived time for 1st ANC visit						
	≤16 weeks	90(28.1)	203(63.4)	1.0	1.0	
	> 16 weeks	17(5.3)	10 (3.1)	3.8(1.69–8.70)	3.9(1.61–9.76)^a^	0.003

^a^Statistical significant at 5% alpha level

## Discussion

For many of the essential interventions in ANC, it is crucial to have early identification of underlying conditions. The first ANC visit should be as early as possible in pregnancy [10]. However, evidence in Ethiopia indicated that delayed ANC initiation time among pregnant mothers was high [18–21] .In our study,one third 107 (33.4%) of respondents were delayed to start their first ANC visit with in the first 16 weeks of pregnancy. This finding is higher as compared to a study done in Bengazie (27%, 2007) [22] . This might be due to difference in educational status of mothers between Bengazie and ours. But this finding is comparatively lower than the findings studied in Ndola (68.6%) and Mpongwe (72%) districts of Zambia [23] and South East Tanzania (81.5%) [24], EDHS,2011(89%) [25], Jimma University Specialized Hospital (60.1%) [26], Kembata tembaro Zone (68.6%) [27] and Dembech district of East Gojjam Zone,North west Ethiopia (94.2%) [28] .The possible explanation for this observed difference might be due to study population composition in which proportion of mothers residing in rural areas of current study were lower than other studies.

The finding also showed a significant association of residence and delay on timely initiation of ANC. Those mothers who were residing in rural areas were 2.8 times more likely delayed than urban mothers. Among all respondents, 56% of rural and 27% of urban residents were start ANC after 16 weeks of gestation. Ethiopian demographic and health survey, 2011, revealed that urban mothers made their first visit earlier (4.4 months) than rural mothers (5.5 months) [25]. This finding is also in agreement with the study done in Vietnam in which rural mothers were attained ANC latter and used fewer visits [29].

The possible reason might be better educational status of urban mothers than rular mothers. Because, in our study, 60% of rural mothers were not attaned formal education compared with 23% of urban mothers. The other reason might be availability of alternative health care facilities and having a better chance of health information in urban areas than rular areas. How ever, in contrary to our result,there was no significant difference in the proportionof delayed ANC attendance between urban and rular areas of Zambia [23].The reason for this difference might be the presence of more active mobile maternity service in rular than urbans areas of Zambia.

Educational status was found to have significant association with delayed initation of first ANC visit. Those mothers who had no formal education were delay two times more likely than those who had teritiary education. This is consistent with studies done in Gondar, Kembata tembaro zone,Tanzania and Ghana [11, 24, 27, 30] in which women who had lower education or none booked later than those with higher education.The reason might be high chance of exposure for information in case of educated mothers.

Eventhough parity had no association with delayed initiation of ANC in our study, 40.7% of multiparous and 25.9% of nuliparous mothers were start thier first ANCvisit after 16 weeks of gestation.But, it was one of the factors for delayed initiation of ANC in different studies [13, 31, 32].

Intention of pregnancy was significantly associated with delay on initiation of first ANC visit. In this study, women with un planned pregnancy were 3.6 times more likely delayed to initiate first ANC visit than those mothers with planned pregnancy.This finding was inline with a study done in Kembata tembaro Zone [26]. Findings in different studies also in agreement with the association of unplanned pregnancy and delayed initiation of first ANC visit [18, 32–35]. The possible reason might be mothers having intended pregnancy are much cautious and eger to know their pregnancy status and less likely delayed than those who had unintended pregnancy.

Intention of abortion in case of unintended pregnancymight also increased the chance of delayed initiation of ANC.Because, above half 14(56%) of pregnant mothers with unintended pregnancy, who looked for abortion did their first ANC visit after 16 weeks of gestation in our study.

Perception on the right time of ANC initiation was found to be significantly associated with delayed ANC visit. Those mothers who perceived that the right initiation period of first ANC visit is beyond 16 weeks of gestation, were four times delayed than their counter part.

The Possible reason might be decreased exposure for information related to ANC initiation time. Because, in this study only 38(11.8%) of mothers have got information about initiation time of ANC from other persons. Other similar studies also suggested that proper information and advice on pattern of ANC utilization is important to book early [33]; where as, not knowing the right gestational age at which to start the first antenatal care visit was the commonest reason for late ANC attendance [36]. So, appropriate perception of the initial ANC visit was a factor for an early ANC visit. This is inagreement with a study done in Gondar University Hospital [30].

### Limitation of the study

The study design is not strong enough to identify determinant factors.

## Conclusion

The magnitude of delay on initiation of first ANC visit was still high but it is lower than studies conducted in other areas of Africa and Ethiopia.The time of initiation was ranges from first timester to third trimester and the mean gestational age on initation of first ANC was arround second trimester. According to this study residence, educational status, intention of pregnancy and perception on initiation time of ANC were influencing mothers on timely initiation of first ANC visit. Awarness creation towards timely initiation of first ANC visit and family planning utilization to prevent unplanned pregnancy should be strengthen by Zonal health department in the community specially for the reproductive age group and rural residents. Ministry of education should also improve women education by strengthening adult education in the community. Further study is recommended in the rural community.
